# Stevens-Johnson syndrome and toxic epidermal necrolysis: A systematic review of PubMed/MEDLINE case reports from 1980 to 2020

**DOI:** 10.3389/fmed.2022.949520

**Published:** 2022-08-24

**Authors:** Liqin Wang, Sheril Varghese, Fatima Bassir, Ying-Chin Lo, Carlos A. Ortega, Sonam Shah, Kimberly G. Blumenthal, Elizabeth J. Phillips, Li Zhou

**Affiliations:** ^1^Division of General Internal Medicine and Primary Care, Department of Medicine, Harvard Medical School, Brigham and Women’s Hospital, Boston, MA, United States; ^2^School of Medicine, Vanderbilt University, Nashville, TN, United States; ^3^Harvard Medical School, Dana-Farber Cancer Institute, Boston, MA, United States; ^4^Division of Rheumatology, Allergy, and Immunology, Harvard Medical School, Massachusetts General Hospital, Boston, MA, United States; ^5^Division of Infectious Disease, Department of Medicine, Vanderbilt University Medical Center, Nashville, TN, United States

**Keywords:** toxic epidermal necrolysis, Stevens-Johnson syndrome, drug-related side effects and adverse reactions, case report, review literature

## Abstract

**Background:**

Stevens-Johnson syndrome (SJS) and toxic epidermal necrolysis (TEN) are rare, life-threatening immunologic reactions. Prior studies using electronic health records, registries or reporting databases are often limited in sample size or lack clinical details. We reviewed diverse detailed case reports published over four decades.

**Methods:**

Stevens-Johnson syndrome and toxic epidermal necrolysis-related case reports were identified from the MEDLINE database between 1980 and 2020. Each report was classified by severity (i.e., SJS, TEN, or SJS-TEN overlap) after being considered a “probable” or “definite” SJS/TEN case. The demographics, preconditions, culprit agents, clinical course, and mortality of the cases were analyzed across the disease severity.

**Results:**

Among 1,059 “probable” or “definite” cases, there were 381 (36.0%) SJS, 602 (56.8%) TEN, and 76 (7.2%) SJS-TEN overlap cases, with a mortality rate of 6.3%, 24.4%, and 21.1%, respectively. Over one-third of cases had immunocompromised conditions preceding onset, including cancer (*n* = 194,18.3%), autoimmune diseases (*n* = 97, 9.2%), and human immunodeficiency virus (HIV) (*n* = 52, 4.9%). During the acute phase of the reaction, 843 (79.5%) cases reported mucous membrane involvement and 210 (19.8%) involved visceral organs. Most cases were drug-induced (*n* = 957, 90.3%). A total of 379 drug culprits were reported; the most frequently reported drug were antibiotics (*n* = 285, 26.9%), followed by anticonvulsants (*n* = 196, 18.5%), analgesics/anesthetics (*n* = 126, 11.9%), and antineoplastics (*n* = 120, 11.3%). 127 (12.0%) cases reported non-drug culprits, including infections (*n* = 68, 6.4%), of which 44 were associated with a mycoplasma pneumoniae infection and radiotherapy (*n* = 27, 2.5%).

**Conclusion:**

An expansive list of potential causative agents were identified from a large set of literature-reported SJS/TEN cases, which warrant future investigation to understand risk factors and clinical manifestations of SJS/TEN in different populations.

## Introduction

Stevens-Johnson syndrome and toxic epidermal necrolysis (SJS/TEN), characterized by the detachment of the epidermis and mucous membrane, are rare severe cutaneous adverse reactions. SJS/TEN can be life-threatening, with mortality rates between 4.8% and 14.8% (1). Based on the degree of skin detachment, SJS/TEN can be classified into SJS, SJS-TEN overlap, and TEN (2). SJS is defined as skin involvement of <10%; TEN is defined as skin involvement of >30%; SJS-TEN overlap is defined as 10−30% skin involvement. The estimated incidences of SJS, SJS/TEN, and TEN in the United States are 9.2, 1.6, and 1.9 per million adults, respectively (1, 3).

The low incidence among patient populations has created unique challenges in elucidating the epidemiology and etiology of SJS/TEN. The optimal medical management of SJS and TEN demands prompt recognition and immediate withdrawal of the causative drugs to alter the course of the reaction and potentially evade mortality. Most prior SJS/TEN studies report findings based on small sample sizes and do not reflect the heterogeneity of the patient population affected by SJS/TEN, minimizing the generalizability of the findings (4, 5). While common causative agents are increasingly identified, little is known about uncommon and non-drug factors that are highly associated with SJS/TEN (6). For example, in two large European case-control studies, fewer than a dozen medications accounted for half of the analyzed SJS/TEN cases (7, 8). Without an exhaustive list of diverse culprits, efforts to promptly withdraw causative agents are inhibited, leading to increased morbidity and mortality.

Several studies attempted to circumvent these limitations by extracting data from electronic health records (EHRs) and large repositories (9–14). For example, Micheletti et al. (11) performed a retrospective cohort study, notably collecting data across 18 United States medical centers and identified 377 SJS/TEN cases from EHRs. Blumenthal et al. used the EHR allergy list to identify over 700 patients with SJS/TEN (11). Similar studies have taken place in Asia, identifying hundreds of patients with SJS/TEN using EHRs or registry databases (15, 16). As a result of such regional studies, it is evident that there are ethnic and regional disparities in the incidence of SJS/TEN that may arise from variation in genetics or regional medical practices (5). SJS/TEN cases have also been identified from post-marketing surveillance adverse events reporting systems; however, such cases often lack stringent SJS/TEN definitions, clinical details, and clear causal associations between drugs and adverse events (17, 18).

Considering the rarity of SJS/TEN and the challenges of collecting validated SJS/TEN cases from EHRs or registry databases, case reports from the literature can be a rich source of information to study SJS and TEN. An appreciable number of case reports have been published to highlight suspected culprit agents and effective care for SJS/TEN cases. Case reports from the literature serve to relay clinical knowledge on a case-by-case basis; they are a unique source of detailed medical information for conditions with low prevalence and undefined care. Although several studies have used case reports to study specific culprit agents (19, 20), currently, no research to our knowledge has contextualized and extrapolated significant trends across all case reports. Cognizant of the logistical barriers to evidence-based research and the need to develop a deep understanding of the etiology, optimal care, and patient outcomes of SJS/TEN, this study seeks to conduct a systematic review of case reports from the literature. By amassing data across case reports from an up-to-date database, PubMed/MEDLINE, we aim to assemble a large, diverse SJS/TEN sample set to comprehensively describe the causative agents, trends over time, differences across disease severity, and patient outcomes.

## Methods

### Data sources and collection

We queried PubMed/MEDLINE on 23 March 2021 to retrieve case reports related to SJS and TEN published between 1 January 1980 and 31 December 2020 (see Table 1).

**TABLE 1:** PubMed/MEDLINE query to retrieve case reports related to Stevens-Johnson syndrome (SJS) and toxic epidermal necrolysis (TEN).

PubMed/MEDLINE search strategy	No. of retrieved reports
(“Case Reports”[pt] OR “Case report”[tiab]) AND ((“toxic”[tiab] AND “epidermal”[tiab] AND “necrolysis”[tiab]) OR (“Steven”[tiab] AND “Johnson”[tiab]) OR (“Lyell”[tiab] AND “Syndrome”) OR (“Stevens-Johnson Syndrome”[MeSH])) AND (“1980/01/01”[PDat]: “2020/12/31”[PDat]) AND (“English”[LA])	1982

The publication date was defined as the date that records were made publicly available in PubMed/MEDLINE regardless of the journal issue date of the case reports.

### Inclusion and exclusion criteria

We included case reports that were written in English with full-text available. We excluded duplicated case reports and any case reports describing more than one SJS/TEN case as the cases in those reports were often discussed in an aggregated manner and more likely to have limited clinical details. However, we included case reports that mentioned multiple cases yet only discussed one SJS/TEN case in detail. We also excluded cases that did not provide enough details about the acute phase of the reaction, such as case reports focused on SJS/TEN sequalae without describing the potential cause, the care received, or the disease progression.

### Annotation process and schema

**FIGURE 1:**
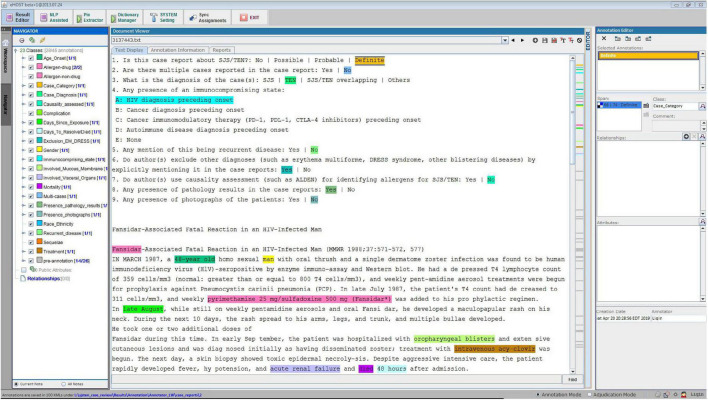
Case report annotation environment.

After collecting the full-text case reports in PDF format, we converted them into text files for annotation. To facilitate manual review, we adopted an open-source annotation tool (i.e., eHOST) to support the extraction of relevant information from the case reports (Figure 1; 21). Each report was annotated by two researchers, and any conflict between the two annotators was resolved by reaching consensus or by a third reviewer. The annotation task was based on an annotation schema, with annotators identifying relevant text in the case reports and assigning the text to a class defined in the schema. We manually defined the schema to cover a broad range of topics for analysis, including age of onset, gender, race/ethnicity, preexisting conditions, involvement of visceral organs and mucous membranes during the acute phase, drug and/or non-drug culprit agents, treatments received, and mortality status.

We also developed 9 questions (see Table 2) and inserted them at the beginning of each report’s text to extract additional information from the case report. The questions included whether the case report was about SJS/TEN and whether there were multiple cases examined. If the case report was related to SJS/TEN, the annotators continued to answer the remaining questions regarding the severity level of the diagnosis (i.e., SJS, TEN, SJS-TEN overlap, or others), whether pathology results were reported, and whether patient photos were provided. They also judged whether there was a recurrence of SJS/TEN and if the authors used casualty assessment [e.g., algorithm of drug causality for epidermal necrolysis [ALDEN] (22)] for identifying culprit agents.

**TABLE 2:** Questions answered by annotators for each case report.

(1) Is this case report about SJS/TEN? No | Possible | Probable | Definite (2) Are there multiple cases reported in the case report: Yes | No (3) What is the diagnosis of the case(s): SJS | TEN | SJS/TEN overlapping | Others (4). Any presence of an immunocompromising state: A: HIV diagnosis preceding onset B: Cancer diagnosis preceding onset C: Cancer immunomodulatory therapy (PD-1, PD-L1, CTLA-4 inhibitors) preceding onset D: Autoimmune disease diagnosis preceding onset E: None (5) Any mention of this being recurrent disease: Yes | No (6) Do author(s) exclude other diagnoses (such as erythema multiforme, DRESS syndrome, other blistering diseases) by explicitly mentioning it in the case reports: Yes | No (7) Do author(s) use causality assessment (such as ALDEN) for identifying allergens for SJS/TEN: Yes | No (8) Any presence of pathology results in the case reports: Yes | No (9) Any presence of photographs of the patients: Yes | No

### Data cleaning

After applying the inclusion and exclusion criteria, we formed a final set of SJS/TEN cases to be included in the analyses. Due to variability in how information was reported, we manually mapped the annotations to standardized terms; for example, “Bactrim,” “TMX-SMZ,” and “co-trimoxazole” were mapped to “trimethoprim-sulfamethoxazole.” Next, we determined the drug and non-drug class for individual allergens based on the First Databank drug classification and manual expert review. We converted the annotations into numerical or categorical values before including them for analysis. Ages were converted to years; if the patient’s age was less than 12 months, it was coded as 0 year. Race, preconditions, and drug and non-drug allergens were manually reviewed and grouped. Mortality and mucous membrane and visceral organ involvement were converted to binary variables.

### Statistical analysis

We described patient demographics and clinical characteristics by severity (i.e., SJS, TEN, and SJS-TEN overlap). Categorical variables are presented as numbers (percentage) and continuous variables are reported as median ± inter-quartiles range. Continuous variables were compared using one-way analysis of variance (ANOVA) test for normally distributed variables or Kruskal-Wallis test for non-normally distributed variables. Categorical variables were compared using Chi-square test. *Post hoc* test was applied after a significant ANOVA, Kruskal-Wallis or Chi-square test, adjusted by Bonferroni correction. The distribution of the cases was analyzed by publication year, severity type, and allergens. Statistical analyses were completed using R software, version 4.0.4 (R Foundation for Statistical Computing).

## Results

### Identification of Stevens-Johnson syndrome and toxic epidermal necrolysis case reports from the literature

**FIGURE 2:**
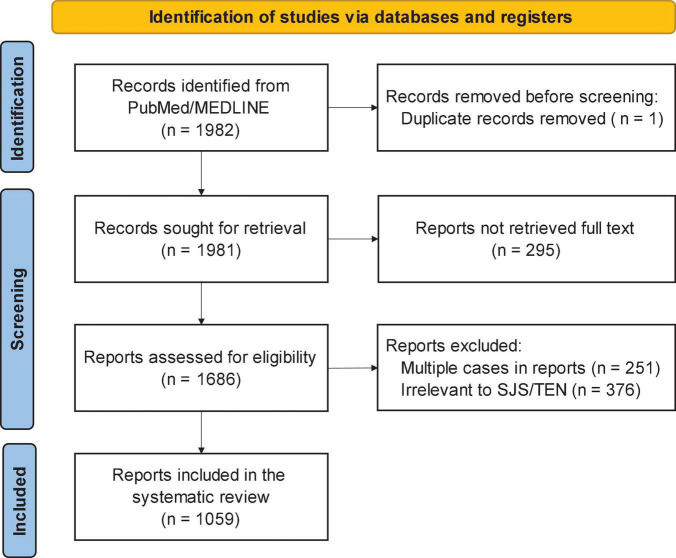
PRISMA flow diagram for choosing Steven Johnson syndrome (SJS) and toxic epidermal necrolysis (TEN) case reports for analysis.

Figure 2 shows the PRISMA diagram for choosing case reports to be included in the analysis (23). The PubMed query returned a total of 1,982 case reports. We excluded 1 duplicate report, 295 reports without full text, 251 multi-case reports, and 376 reports that were irrelevant or did not contain sufficient clinical details of SJS/TEN. In total, 1,059 case reports met the inclusion criteria, which were composed of 381 (36.0%) SJS, 602 (56.8%) TEN, and 76 (7.2%) SJS-TEN overlap cases. Of included reports, 538 (50.8%) included pathology results and 700 (66.1%) contained photographs.

### Publication trends

**FIGURE 3:**
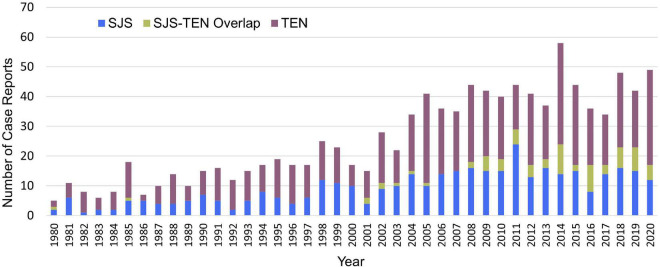
Distribution of Stevens-Johnson syndrome (SJS), toxic epidermal necrolysis (TEN) and SJS-TEN overlap case reports from PubMed/MEDLINE by publication year.

Figure 3 shows the distribution of SJS, TEN, and SJS-TEN overlap cases by publication year. All cases were published between 1980 and 2020 with 273 (25.8%) cases published before 2000. The number of case reports peaked in 2014 with a total of 58 case reports.

### Demographics and clinical characteristics of Stevens-Johnson syndrome and toxic epidermal necrolysis cases

Table 3 shows the overall demographics and clinical characteristics of the SJS/TEN cases by severity. Approximately 52.6% (*n* = 557) of all included cases were female. Less than half of the sample with an SJS diagnosis were female, unlike the TEN and SJS-TEN overlap samples (46.1% in SJS, 56.1% in TEN, and 56.6% in SJS-TEN overlap, *p*-Value = 0.007). The majority of cases (*n* = 795, 75.1%) did not report race or ethnicity.

**TABLE 3:** Demographics and clinical characteristics of Stevens-Johnson syndrome (SJS) and toxic epidermal necrolysis (TEN) cases from PubMed/MEDLINE.

Characteristics	Total (*n* = 1,059)	SJS (*n* = 381)	SJS-TEN Overlap (*n* = 76)	TEN (*n* = 602)	*P*-value^a^
Age of onset^b^ (y), median (IQR)	38 (19.75−59)	**32 (15**−**54)**	39 (23−58)	**41 (23**−**60.75)**	<0.001
Gender, female^b^	557 (52.6)	**176 (46.3)**	43 (56.6)	**338 (56.1)**	0.007
Race^b^					0.832
White	105 (9.9)	34 (8.9)	8 (10.5)	63 (10.5)	
Asian	87 (8.2)	28 (7.3)	8 (10.5)	51 (8.5)	
Black	54 (5.1)	22 (5.8)	4 (5.3)	28 (4.7)	
Hispanic	11 (1.0)	5 (1.3)	2 (2.6)	4 (0.7)	
Others^c^	7 (0.7)	3 (0.8)	0 (0.0)	4 (0.7)	
Immunocompromised status					
Cancer	194 (18.3)	61 (16.0)	11 (14.5)	122 (20.3)	0.163
Cancer immunomodulatory therapy (PD-1, PD-L1, CTLA-4 inhibitor)	35 (3.3)	16 (4.2)	2 (2.6)	17 (2.8)	0.473
Autoimmune disease	97 (9.2)	28 (7.3)	8 (10.5)	61 (10.1)	0.31
HIV/AIDS	52 (4.9)	22 (5.8)	1 (1.3)	29 (4.8)	0.256
Pre-conditions					
Infections	201 (19.0)	83 (21.8)	9 (11.8)	103 (17.1)	0.056
Respiratory tract infections	102 (9.6)	48 (12.6)	4 (5.3)	50 (8.3)	0.034
Mycoplasma pneumoniae infections	23 (2.2)	**20 (5.2)**	1 (1.3)	**2 (0.3)**	<0.001
Epilepsy/seizure disorders	102 (9.6)	37 (9.7)	10 (13.2)	55 (9.1)	0.533
Hypertension	92 (8.7)	29 (7.6)	6 (7.9)	57 (9.5)	0.636
Cardiovascular/vascular conditions	54 (5.1)	15 (3.9)	7 (9.2)	32 (5.3)	0.149
Diabetes	54 (5.1)	17 (4.4)	4 (5.3)	33 (5.5)	0.814
Musculoskeletal conditions	52 (4.9)	18 (4.7)	2 (2.6)	32 (5.3)	0.693
Endocrine/hormonal conditions	50 (4.7)	20 (5.2)	3 (3.9)	27 (4.5)	0.849
Psychological conditions	38 (3.6)	13 (3.4)	4 (5.3)	21 (3.5)	0.656
Renal conditions	34 (3.2)	12 (3.1)	1 (1.3)	21 (3.5)	0.73
Substance use	28 (2.6)	9 (2.4)	2 (2.6)	17 (2.8)	0.954
Gastrointestinal conditions	22 (2.1)	3 (0.8)	1 (1.3)	18 (3.0)	0.053
Respiratory conditions (e.g., chronic obstructive pulmonary disease)	20 (1.9)	10 (2.6)	1 (1.3)	9 (1.5)	0.388
Other^d^	27 (2.5)	8 (2.1)	2 (2.6)	17 (2.8)	−
Clinical characteristics during the acute phase					
Involvement of mucous membrane	842 (79.5)	**333 (87.4)**	**70 (92.1)**	**439 (72.9)**	<0.001
Involvement of visceral organs	210 (19.8)	**56 (14.7)**	18 (23.7)	136 (22.6)	0.007
Mortality	187 (17.6)	**24 (6.3)**	16 (21.1)	**147 (24.4)**	<0.001
Medications listed as causative agents, No. (%)	956 (90.3)	**319 (83.7)**	74 (97.4)	**563 (93.5)**	<0.001
1^e^	781 (73.7)	266 (69.8)	63 (82.9)	451 (74.9)	0.36
2	111 (10.5)	38 (10.0)	9 (11.8)	64 (10.6)	
3	39 (3.7)	9 (2.6)	1 (1.3)	29 (4.8)	
4	16 (1.5)	5 (1.3)	1 (1.3)	10 (1.7)	
5 or more	10 (0.9)	1 (0.3)	0 (0)	9 (1.5)	
Non-drug listed as causative agents, No. (%)	127 (12.0)	**71 (18.6)**	6 (7.9)	**50 (8.3)**	<0.001
Non-drug causative agents only	81 (7.6)	52 (13.6)	2 (2.6)	27 (4.5)	−
1	73 (6.9)	48 (12.6)	2 (2.6)	23 (3.8)	
2 or more	8 (0.8)	4 (1.0)	0 (0)	4 (0.7)	
Combined with drug causative agents	46 (4.3)	19 (5.0)	4 (5.3)	23 (3.8)	

IQR, interquartile range; HIV, human immunodeficiency virus; AIDS, acquired immunodeficiency syndrome.

For continuous variables, the number (percentage) in bold indicates a significant difference between the cells detected by Dunn’s post hoc test. For categorical variables, the number (percentage) in bold indicates a significant adjusted residual for that cell (meaning that there were significantly more or fewer cases than what would be expected by chance).

^a^*P*-values were provided based on Kruskal-Wallis test for the continuous variable (age of onset) and Chi-square test for categorical variables.

^b^The number of missing cases (age of onset = 7; gender = 3; race = 795).

^c^Includes native American, Pacific Islander, mixed race.

^d^Includes skin/cutaneous (n = 9), hereditary (n = 8), and neurological conditions (n = 10).

^e^The numbers were calculated based on the annotated medications. Due to the variation of medications, this numbers can be under-counted.

Out of all 1,059 cases, 194 patients had a cancer diagnosis, 35 patients were receiving cancer immunomodulatory therapy (PD-1, PD-L1, CTLA-4 inhibitors), 97 patients had an autoimmune disease diagnosis (i.e., systemic lupus erythematosus, rheumatoid arthritis, psoriasis), and 52 patients presented with an HIV diagnosis preceding onset. About 20% and 16% of patients diagnosed with TEN and SJS, respectively, were diagnosed with cancer. 2.0% of TEN cases and 2.6% of SJS cases were receiving cancer therapy at the time of their SJS/TEN diagnoses. Altogether, 7−10% of cases in all groups were documented to have at least one autoimmune disease.

Among all the SJS/TEN cases, infections were the most common preconditions prior to SJS/TEN onset (*n* = 201, 19.0%). The presentation of infections is highest among SJS cases (21.8%) compared to TEN (17.1%) and SJS-TEN overlap (11.8%) cases. This pattern applies to respiratory tract infections and mycoplasma pneumonia infections, while the later one also shows a significant difference across the three-severity groups (*p*-Value < 0.001). Other less common preconditions include epilepsy/seizure disorders (*n* = 102, 9.6%), hypertension (*n* = 92, 8.7%), cardiovascular conditions (*n* = 54, 5.1%), diabetes (*n* = 54, 5.1%), musculoskeletal conditions (*n* = 52, 4.9%), and endocrine/hormonal conditions (*n* = 50, 4.7%).

We also extracted data from the case reports regarding the acute phase of SJS/TEN. The majority of cases (*n* = 842, 79.5%) reported involvement of mucosal membranes, including the oropharynx, conjunctiva, genitalia, and/or anus. The SJS-TEN overlap cases reported the highest percentage of patients with mucosal membrane involvement (92.1%), while TEN cases, the severest of the three diagnoses, reported the lowest rate of mucous membrane involvement (73.0%). 210 (19.8%) cases reported that visceral organs were impacted throughout the diagnosis. Fewer patients in the SJS cohort experienced involvement of visceral organs relative to both SJS-TEN overlap and TEN cases alone (14.7% vs. 23.7% vs. 22.6%, *p*-Value = 0.007).

Approximately 18% (*n* = 187) of patients diagnosed with SJS/TEN did not survive. Case reports with a TEN diagnosis reported the highest mortality relative to patients diagnosed with SJS-TEN overlap syndrome and SJS (TE*N* = 24.4%, SJS-TEN overlap = 21.1%, SJS = 6.3%, *p*-Value < 0.001).

### Causative agents of Stevens-Johnson syndrome and toxic epidermal necrolysis cases

Of all cases, 957 (90.3%) implicated medications as the cause of the diagnoses. 781 (73.7%) cases reported a single medication as the culprit. More TEN and SJS-TEN overlap cases were caused by drug allergens compared to SJS cases (93.7% for TEN, 97.4% for SJS-TEN overlap, and 83.7% for SJS). 127 (12.0%) cases implicated non-drug culprit agents, of which 46 were concurrently exposed to drug agents. 16 (1.5%) cases did not report the cause of the reaction.

Table 4 shows the number of SJS/TEN cases caused by drug and non-drug culprits across the spectrum of severity. A total of 379 drugs were associated with the SJS/TEN cases, more than half of which (*n* = 226, 59.6%) were associated with only one case. Phenytoin, trimethoprim-sulfamethoxazole, carbamazepine, lamotrigine, allopurinol, acetaminophen, amoxicillin, ibuprofen, phenobarbital, and vancomycin were the most reported drugs, each associated with over twenty SJS/TEN cases. The most frequently suspected drug class was antibiotics (*n* = 285, 26.9%), which includes sulfonamides (*n* = 108, 10.2%), penicillins (*n* = 60, 5.7%), and quinolones (*n* = 35, 3.3%) (Table 4). Antibiotics were reported as the causative agent in TEN cases (30.1%) slightly more than in SJS (22.1%) and SJS-TEN overlap (26.3%) cases primarily due to sulfonamides. Quinolones were reported to cause the fewest number of SJS cases (1.6%) relative to TEN (4.2%) and SJS-TEN overlap (5.2%) cases. Anticonvulsants, including phenytoin, carbamazepine, lamotrigine, and valproate, are also associated with a significant number of SJS/TEN cases (*n* = 196, 18.5%) and were reported to cause a greater amount of TEN (19.4%) and SJS-TEN overlap (25.0%) cases compared to SJS cases (15.7%). Analgesics/anesthetics were also commonly reported, with a total of 126 (11.9%) cases, 93 of which were associated with non-steroidal anti-inflammatory drugs (NSAIDs). Antineoplastics were reported in 120 (11.3%) SJS/TEN cases. Detailed medications under each category as well as the number of associated SJS/TEN cases are reported in Table 5.

**TABLE 4:** Drug and non-drug allergens reported to cause SJS or TEN among reported cases from the literature.

Allergen^a^	Total (*n* = 1,059)	SJS (*n* = 381)	SJS-TEN Overlap (*n* = 76)	TEN (*n* = 602)	*P*-value^b^
Drug Allergen					
*Antibiotics*	285 (26.9)	84 (22.1)	20 (26.3)	181 (30.1)	0.022
Sulfonamides	108 (10.2)	26 (6.8)	5 (6.6)	77 (12.8)	−
Penicillins	60 (5.7)	21 (5.5)	6 (7.9)	33 (5.5)	−
Quinolones	35 (3.3)	6 (1.6)	4 (5.2)	25 (4.2)	−
Macrolides	25 (2.4)	12 (3.2)	1 (1.3)	12 (2.0)	−
Vancomycin	21 (2.0)	2 (0.5)	2 (2.6)	17 (2.8)	−
Tetracycline	11 (1.0)	4 (1.1)	1 (1.3)	6 (1.0)	−
Other antibiotics^a^	72 (6.8)	19 (5.0)	5 (6.6)	48 (8.0)	−
Anticonvulsants	196 (18.5)	60 (15.7)	19 (25.0)	117 (19.4)	0.111
Phenytoin	62 (5.7)	16 (4.2)	3 (3.9)	43 (7.1)	−
Carbamazepine	54 (5.1)	15 (3.9)	8 (10.5)	31 (5.1)	−
Lamotrigine	49 (4.6)	20 (5.2)	2 (2.6)	27 (4.5)	−
Valproate	16 (1.5)	8 (2.1)	2 (2.6)	6 (1.0)	−
Other anticonvulsants	32 (3.0)	10 (2.6)	5 (6.6)	17 (2.8)	−
Analgesics/anesthetics	126 (11.9)	34 (8.9)	14 (18.4)	78 (13.0)	0.031
NSAIDs	93 (8.8)	24 (6.3)	9 (11.8)	60 (10.0)	−
Ibuprofen	23 (2.2)	6 (1.6)	5 (6.6)	12 (2.0)	−
Acetaminophen	24 (2.3)	5 (1.3)	3 (3.9)	16 (2.7)	−
Analgesic/antipyretics, non-salicylate	37 (3.5)	10 (2.6)	6 (7.9)	21 (3.5)	−
Other	5 (0.3)	1 (0.3)	1 (1.3)	3 (0.5)	−
Antineoplastics	119 (11.2)	42 (11.0)	10 (13.2)	67 (11.1)	0.858
Systemic enzyme inhibitors (e.g., imatinib)	24 (2.3)	16 (4.2)	1 (1.3)	7 (1.2)	−
Antimetabolites (e.g., methotrexate)	19 (1.8)	3 (0.8)	3 (3.9)	13 (2.2)	−
Alkylating agents (e.g., cyclophosphamide)	15 (1.4)	3 (0.8)	1 (1.3)	11 (1.8)	−
Immunotherapy checkpoint inhibitor combination (nivolumab)	12 (1.1)	4 (1.0)	0 (0)	8 (1.3)	−
Immunomodulator agents (e.g., lenalidomide)	11 (1.0)	6 (1.6)	1 (1.3)	4 (0.7)	−
Other antineoplastics	50 (4.7)	12 (3.1)	4 (5.3)	34 (5.6)	−
Antiarthritics	48 (4.5)	14 (3.7)	6 (7.9)	28 (4.7)	0.265
Xanthine oxidase inhibitors (allopurinol)	45 (4.2)	14 (3.7)	6 (7.9)	25 (4.2)	−
Antivirals	34 (3.2)	14 (3.7)	3 (3.9)	17 (2.8)	0.71
HIV-specific antivirals (e.g., nevirapine)	25 (2.4)	12 (3.1)	0 (0)	13 (2.2)	−
Gastrointestinal drugs (e.g., sulfasalazine)	34 (3.2)	8 (2.1)	2 (2.6)	24 (4.0)	0.251
Psychotherapeutic drugs	25 (2.4)	12 (3.1)	1 (1.3)	12 (2.0)	0.419
Antidepressant	11 (1.0)	6 (1.6)	0 (0)	5 (0.8)	−
Anti-Infectives	24 (2.3)	12 (3.1)	0 (0)	12 (2.0)	0.191
Antimalarial drugs	20 (1.9)	11 (2.9)	0 (0)	9 (1.5)	−
Antifungals	20 (1.9)	7 (1.8)	0 (0)	13 (2.2)	0.426
Cardiovascular drugs	27 (2.5)	8 (2.1)	0 (0)	19 (3.2)	0.203
Diuretics	17 (1.6)	7 (1.8)	1 (1.3)	9 (1.5)	0.897
Vitamin/herb	15 (1.4)	8 (2.1)	0 (0)	7 (1.2)	0.267
Hormones	14 (1.3)	5 (1.3)	0 (0)	9 (1.5)	0.561
Glucocorticoids	11 (1.0)	3 (0.8)	0 (0)	8 (1.3)	−
Biologicals/vaccine	10 (0.9)	6 (1.6)	1 (1.3)	3 (0.5)	0.222
Diagnostic (contrast medium)	10 (0.9)	3 (0.8)	2 (2.6)	5 (0.8)	0.287
Chemotherapy rescue/antidote agents	8 (0.8)	3 (0.8)	0 (0)	5 (0.8)	0.73
Antithrombotic agents	8 (0.8)	2 (0.5)	0 (0)	6 (1.0)	0.518
Cough/cold preparations	6 (0.6)	2 (0.5)	0 (0)	4 (0.7)	0.761
Immunosuppressants	6 (0.6)	5 (1.3)	0 (0)	1 (0.2)	0.052
Non-drug Allergen					
Infection	68 (6.4)	**51 (13.4)**	2 (2.6)	**15 (2.5)**	<0.001
Mycoplasma pneumonia infection	44 (4.2)	38 (10.0)	2 (2.6)	4 (0.7)	−
Radiotherapy	27 (2.5)	11 (2.9)	2 (2.6)	14 (2.3)	0.861
Chemical substance	9 (0.8)	5 (1.3)	1 (1.3)	3 (0.5)	0.36
Others	25 (2.4)	4 (2.6)	1 (1.3)	20 (3.3)	−

NSAIDs, non-steroidal anti-inflammatory drugs; HIV, human immunodeficiency virus.

The number (percentage) in bold indicates a significant adjusted residual for that cell (meaning that there were significantly more or fewer cases than what would be expected by chance).

^a^The detailed allergen included in each category could be found in the Tables 5, 6.

^b^*P*-values were provided based on Chi-square test for categorical variables.

**TABLE 5:** Drug category, drug type, and allergen with case count.

Drug Category	Drug Type	Specific Allergen (Number of SJS/TEN Cases)^a^
Antibiotics	Sulfonamides	Trimethoprim/sulfamethoxazole (54), sulfonamides (9), cephalexin (7), ceftriaxone (5), cefotaxime (5), sulfadiazine (5), Sulfamethoxazole (3), sulfadoxine (3), ceftazidime (3), cefuroxime (2), sulfacetamide (2), cefazolin (2), sulfa drugs (1), cefepime (1), cefozopran (1), cefsulodin (1), ceftizoxime (1), cefixime (1), cephradine (1), maxipime (1), sulfa antibiotic therapy (1), sulfapyridine (1), sulfisoxazole (1), cefamandole (1), cefaclor (1), cefotiam hydrochloride (1)
	Penicillins	Amoxicillin (24), ampicillin (12), penicillin (8), amoxicillin/clavulanic acid (7), piperacillin/tazobactam (5), oxacillin (2), cloxacillin (2), flucloxacillin (2), amoxycillin (1), ampicillin/sulbactam (1), coamoxiclav (1)
	Quinolones	Ciprofloxacin (11), levofloxacin (10), moxifloxacin (4), norfloxacin (3), ofloxacin (3), lomefloxacin (1), sparfloxacin (1), tosufloxacin (1), trovafloxacin (1)
	Macrolides	Azithromycin (13), erythromycin (7), clarithromycin (3), roxithromycin (2)
	Vancomycin	Vancomycin (21)
	Tetracycline	Doxycycline (7), tetracycline (2), tigecycline (1), minocycline (1)
	Other antibiotics	Antibiotics therapy (7), trimethoprim (7), thalidomide (7), meropenem (6), teicoplanin (5), rifampin (4), gentamicin (3), amikacin (3), cephalosporin (3), nitrofurantoin (3), tobramycin (3), clindamycin (3), aztreonam (2), metronidazole (2), ethambutol (2), rifaximin (2), lincomycin (2), mupirocin (1), anti-tuberculosis medication (1), antibiotics (1), bacitracin (1), cephem (1), chloramphenicol (1), cilastatin (1), cycloserine (1), dapsone (1), ertapenem (1), furazolidone (1), imipenem (1), oral medication for an upper respiratory tract infection (1), pristinamycin (1), pyrazinamide (1), rifabutin (1), streptomycin (1), telithromycin (1)
Anticonvulsants		Phenytoin (61), carbamazepine (54), lamotrigine (49), valproate (15), oxcarbazepine (7), levetiracetam (5), zonisamide (4), antiepileptic drugs (3), clobazam (3), lacosamide (1), anticonvulsant (1), cannabidiol (1), felbamate (1), gabapentin (1), anticonvulsants (1), nitrazepam (1), phenylhydantoin (1), rufinamide (1), tetrazepam (1), trazepam (1), valproic acid (1)
Analgesics/anesthetics	Anagelsics/antipyretics/non-salicylates	Acetaminophen (36), phenacetin (1), dipyrone (1)
	NSAIDs	Ibuprofen (23), etoricoxib (6), acetylsalicylic acid (6), diclofenac (5), non-steroidal anti-inflammatory drug (5), naproxen (5), benoxaprofen (4), mefenamic acid (4), anti, inflammatory drug (3), celecoxib (3), metamizole (3), nimesulide (3), salicylamide (2), diacerein (2), piroxicam (2), ketoprofen (2), oxaprozin (2), indomethacin (2), fenbufen (1), isoxicam (1), loxoprofen (1), Mesalazine (1), methampyrone (1), diflunisal (1), oxyphenbutazone (1), rofecoxib (1), salicylates (1), sulindac (1), valdecoxib (1), diclofenac/serratiopeptidase (1), aceclofenac (1), etofenamate (1), Etodolac (1)
	Other analgesics/anesthetics	Analgesics (1), codeine (1), mepivacaine (1), isopropylantipyrin/arylisopropylacetoureid/phenacetinum (1), acetaminophen/oxycodone (1)
Antineoplastics	Alkylating agents	Cyclophosphamide (4), temozolomide (4), chlorambucil (3), cisplatin (1), carboplatin (1), ifosfamide (1), mechlorethamine (1)
	Antimetabolites	Methotrexate (13), gemcitabine (2), pemetrexed (2), capecitabine (1), cytosine arabinoside (1)
	Immunomodulator agents	Lenalidomide (9), everolimus (1), levamisole (1)
	Immunotherapy checkpoint inhibitor combination	Nivolumab (12)
	Systemic enzyme inhibitors	Imatinib (9), osimertinib (3), afatinib (2), sunitinib (2), sorafenib (2), ribociclib (2), vandetanib (1), bortezomib (1), gefitinib (1), Masitinib (1)
	Other antineoplastics	Vemurafenib (8), pembrolizumab (6), mogamulizumab (6), docetaxel (3), cetuximab (3), fulvestrant (2), Ipilimumab (2), vincristine (2), premetrexed/cisplatin (2), letrozole (2), etoposide (2), ofatumumab (1), paclitaxel (1), pd1 inhibitor (1), atezolizumab (1), peplomycin (1), procarbazine (1), rituximab (1), rituximab/bendamustine (1), tamoxifen (1), actinomycin (1), vinorelbine (1), cobimetinib (1), dactinomycin (1), brentuximab vedotin (1), denileukin diftitox (1), enfortumab vedotin (1), etoposide/cisplatin (1), l-asparaginase (1), bleomycin (1)
Antiarthritics	Xanthine oxidase inhibitors	Allopurinol (45)
	Other antiarthritics	Leflunomide (2), penicillamine (1)
Antivirals	HIV-specific antivirals	Nevirapine (17), abacavir (2), efavirenz (2), stavudine (2), zidovudine (2), indinavir (1), darunavir (1), emtricitabine/tenofovir (1), nelfinavir (1)
	Other antivirals	Lamivudine (4), acyclovir (4), oseltamivir (3), adefovir (1), 18 drugs for encephalitis (1)
Gastrointestinal drugs		Sulfasalazine (10), omeprazole (5), ranitidine (5), lansoprazole (3), famotidine (2), hyoscyamine (1), cimetidine (1), dimenhydrinate (1), donnatal (1), glycerin (1), h2 antagonist (1), lactulose (1), pantoprazole (1), prochlorperazine (1), promethazine (1), rabeprazole (1), scopolamine (1)
Psychotherapeutic drugs	Antidepressant	Fluoxetine (2), mirtazapine (2), amoxapine (1), fluvoxamine (1), venlafaxine (1), duloxetine (1), paroxetine (1), sertraline (1), bupropion (1)
	Other psychotherapeutic drugs	Chlorpromazine (3), lithium (2), paliperidone (1), armodafinil (1), benzodiazepines (1), chlordiazepoxide (1), chlormezanone (1), haloperidol (1), modafinil (1), oxazepam (1), thioridazine (1)
Anti-infectives	Antimalarial drugs	Sulfadoxine/pyrimethamine (11), chloroquine phosphate (7), pyrimethamine (3), mefloquine (2), hydroxychloroquine (1), proguanil (1)
	Other anti-infectives	Atovaquone (2), ivermectin (2), pentamidine (1)
Antifungals		Fluconazole (8), voriconazole (3), terbinafine (3), griseofulvin (2), caspofungin (1), amphotericin B (1), itraconazole (1), nystatin (1)
Cardiovascular drugs		Captopril (3), minoxidil (3), carvedilol (2), hydralazine (2), vasoprotectors (1), rosuvastatin (1), atropine sulfate (1), irbesartan (1), nitroprusside (1), phenylephrine (1), ramipril (1), atorvastatin (1), sildenafil (1), timolol (1), vasodilators (1), amiodarone (2), amlodipine (2), nitroglycerin (1), diltiazem (1), dronedarone (1), isosorbide dinitrate (1)
Diuretics		Furosemide (4), methazolamide (4), acetazolamide (2), hydrochlorothiazide (2), indapamide (2), metolazone (2), bumetanide (1), spironolactone (1)
Vitamin/herb		Herbal medication (7), ayurvedic medication (3), ophiopogonis tuber (1), pyritinol (1), supradyn (1), vitamin b complex (1), traditional Chinese medicine (1), golden health blood purifying tablets (1), moringa oleifera (1)
Hormones	Glucocorticoids	Dexamethasone (7), prednisolone (3), betamethasone (1)
	Other hormones	Danazol (1), gemeprost (1), human chorionic gonadotropin (1), medroxyprogesterone acetate (1), cabergoline (1), clomiphene (1)
Biologicals/vaccine		Vaccine (2), influenza vaccine (2), measles vaccine (1), anthrax (1), hantavirus vaccine (1), MPR vaccine (1), rabies vaccination (1), smallpox vaccine (1), tetanus vaccines (1), varicella-zoster virus vaccine (1), yellow fever vaccine (1)
Diagnostic (contrast medium)		Contrast medium (9), diatrizoate meglumine-diatrizoate sodium (1), cardiac catheterization dye (1)
Chemotherapy rescue/antidote agents		Amifostine (5), mesna (1), leucovorin (1), folinic acid (1)
Antithrombotic agents	Anticoagulants	Warfarin (3), warfarin potassium (1), heparin (1), dabigatran (1)
	Antiplatelet drugs	Acetylsalicylic acid/dipyridamole (1), clopidogrel (1), ticlopidine hydrochloride (1)
Cough/cold preparations		Tipepidine (2), phenylpropanolamine (2), pseudoephedrine (2), guaifenesin (1), guaifenesin/pseudoephedrine (1)
Immunosuppressants		Mizoribine (2), tacrolimus (1), azathioprine (1), tocilizumab (1), mycophenolate mofetil (1)
Others		Teriflunomide (1), phenobarbital (22), strontium ranelate (3), ritodrine (3), propylthiouracil (2), adalimumab (2), tranexamic acid (2), glyburide (1), albuterol (1), alfuzosin (1), amphetamine (1), astemizole (1), bromisovalum (1), butalbital (1), carbocisteine (1), cetirizine (1), cocaine (1), contraceptive pills (1), cromoglycate (1), dimercapto-propane sulfonate (1), disulfiram (1), dorzolamide (1), etidronate (1), etretinate (1), fexofenadine (1), glipizide (1), glyphosate (1), immunoglobulin (1), iron protein succinylate (1), lactose (1), latanoprost (1), mancozeb (1), methamphetamine (1), methimazole (1), mifepristone (1), pirenzepine hydrochloride (1), promethazine methylene disalicylate (1), repaglinide (1), suramin (1), titanium silicate (1), some medications (1)

TMP-SMZ, trimethoprim/sulfamethoxazole; NSAIDs, non-steroidal anti-inflammatory drug; MPR, morbilli-parotitis-rubella; SJS/TEN, Stevens-Johnson syndrome and/or toxic epidermal necrolysis.

^a^The case count is reflecting the number of unique cases while some of the cases could have more than one allergen annotations.

Of all SJS/TEN cases, the most common non-drug culprits were infections (*n* = 68, 6.4%), which were reported more frequently to cause SJS (13.4%) compared to TEN (2.5%) and SJS-TEN overlap (2.6%) (*p*-Value < 0.001). Mycoplasma pneumonia infections (*n* = 44, 4.2%) were highest in SJS cases (13.4%) compared to TEN (2.5%) and SJS-TEN overlap cases (2.6%). The second most common non-drug agent implicated in SJS/TEN was radiotherapy, which was reported in 27 SJS/TEN cases; however, many of these cases (*n* = 25) also reported a drug as a causative agent, including anticonvulsants (*n* = 13), antineoplastics (*n* = 4) and chemotherapy rescue drugs (*n* = 3). Chemical substances [e.g., arsenic (24, 25), insecticide (26, 27)] were also reported to cause SJS/TEN. Detailed non-drug culprits as well as the number of associated SJS/TEN cases are reported in Table 6.

**TABLE 6:** Non-drug allergen category and non-drug allergen with case count.

Non-Drug Allergen Category	Allergen Type	Specific Allergen (Number of SJS/TEN Cases)^a^
Infection	Mycoplasma pneumonia infection	*M. pneumoniae* (2), mycoplasma pneumonia infection (40), pneumonia infection (3), upper respiratory infection (2)
	Other infection	Brucella melitensis (1), cytomegalovirus infection (1), dengue virus (1), enterovirus (1), Epstein-Barr virus infection (1), herpes simplex virus (4), influenza B infection (2), mucor infection (1), parvovirus infection (1), pneumonia infection (2), psittacosis (1), respiratory infection (2), staphylococcus septicemia (1), upper respiratory infection (1), varicella-zoster virus (1), varicella infection (1), viral hepatitis type a (1), viral illness (2), yersinia enterocolitica infection (1)
Radiotherapy		Brain radiotherapy (13), cranial radiotherapy (2), radiotherapy (14)
Chemical substance	Chemical compound	Gangliosides (1), s,s-dimethyl cyanocarbonimidodithioate (1), trichloroethylene (1), arsenic (2), Iodine (1), mercury (1), carbamate insecticide (2), organophosphate insecticide (1)
Others	Disease	HIV (1), Hodgkin’s disease (cancer) (1), lupus (1), non-Hodgkin lymphoma (1)
	Others	Acrylonitrile-butadiene-styrene (1), alpha-PVP (1), anhydrous caffeine (1), black widow spider bite (1), burn (1), caffeine (1), cellulose acetate (1), cologne (1), cosmetic cream (1), interleukin-2 (1), oil lamp (1), phototoxic allergy (1), polyvinyl chloride (1), printing inks (1), spirulina (1), sun exposure (1), tanning salon (1), UV-cured inks (1), pregnancy (2), pregnancy (2), bone marrow transplantation (2), stem cell transplantation (2)

SJS/TEN, Stevens-Johnson syndrome and/or toxic epidermal necrolysis; HIV, human immunodeficiency virus.

^a^The case count is reflecting the number of unique cases while some of the cases could have more than one allergen annotations.

### Publication trends of the culprit agents

**FIGURE 4:**
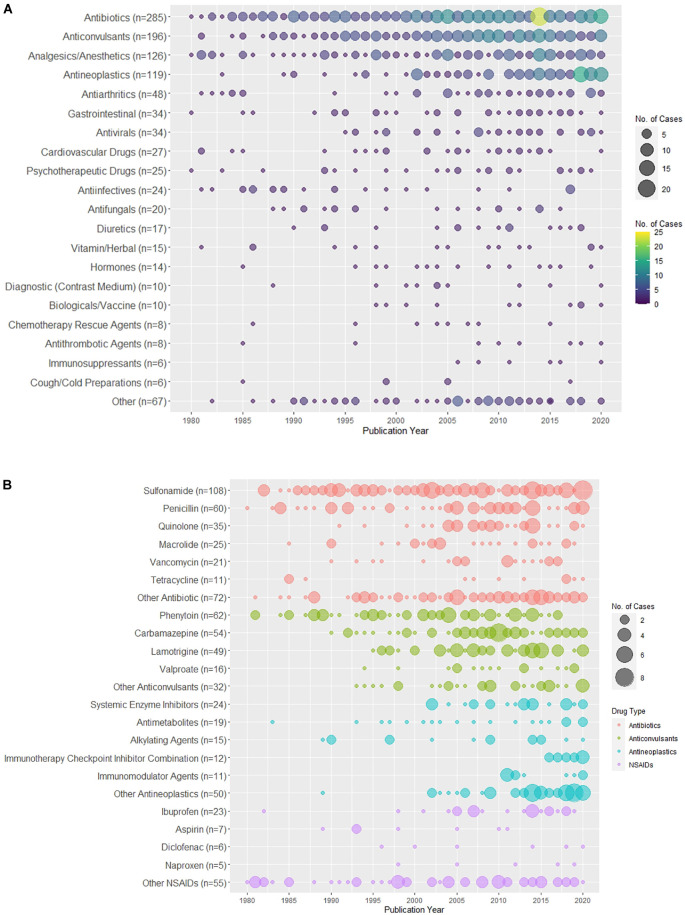
Distribution of drug culprits over the years. **(A)** Distribution of the drug categories of the culprit drugs associated with SJS/TEN over the years. **(B)** Distribution of the culprit drugs of top four common drug categories (antibiotics, anticonvulsants, antineoplastics, non-steroidal anti-inflammatory drugs [NSAIDs]) associated with SJS/TEN over the years.

Figure 4 shows the distribution of drug culprits causing SJS/TEN over time. In particular, Figure 4A shows the distribution of the drug categories, while the distribution of cases caused by specific antibiotics, anticonvulsants, NSAIDs, and antineoplastics over time can be found in Figure 4B.

## Discussion

In the present study, we retrieved a large set of SJS/TEN cases reported in the literature. We described the demographics and clinical characteristics of the cases across the spectrum of severity and identified a variety of drug and non-drug culprits as well as their frequency of being reported over the years. By examining a significant number of SJS/TEN cases from case reports, our investigation overcomes several research limitations and minimizes logistical challenges. Despite the time-consuming nature of annotating over 1,000 case reports, exhaustive manual data extraction ensured the quality of the extracted data. Because it is difficult to conduct robust evidence-based studies and clinical trials that examine the etiology for a rare condition such as SJS/TEN, our review allowed for a broad analysis of clinical cases that were rich with detail. Like current research utilizing EHR or registry data, our data characterizes common causes across many patients and highlights potential agents that have yet to be studied at large, such as herbal medications.

### Publication trends

Overall, the number of published SJS and TEN case reports increased over the past forty years, peaking in 2014. Consistent with other study populations, over half of the cases were female (8, 9, 28). Contrary to the incidence reported in other study populations in this field (1, 9, 29, 30), there were more cases concerning TEN than cases of SJS or SJS-TEN overlap. Although the incidence of TEN is three to four times less than SJS (1, 29), the larger proportion of TEN case reports likely reflects a publication bias for cases with higher clinical severity and complexity. TEN cases reported lower rates of mucous membrane involvement than SJS and SJS-TEN overlap cases, which may be due to a greater degree of skin detachment or underreporting. Also, over 20% of TEN and SJS-TEN overlap cases reported involvement of visceral organs, such as lungs, liver, and kidney, indicating the fatality of the disease and long-term sequalae. With over 600 case reports solely focused on TEN, case reports are an abundant source of information to explore TEN etiology, diagnosis, and treatment.

Publishing trends of the culprit agents reveal that medications classified as antibiotics, anticonvulsants, and analgesics/anesthetics are the dominant culprit agents throughout time. Among these categories, there are several medications that have been repeatedly cited to trigger SJS/TEN. For antibiotics, sulfonamides and penicillins are frequently reported causative agents. Since the 2000s, there has been an increasing number of cases identifying quinolones and vancomycin as the causative agents. Among anticonvulsants, phenytoin is a common causative agent throughout the study period, but from 2015 to 2020, there appears to be a decline in cases citing phenytoin relative to other anticonvulsants. Carbamazepine and lamotrigine, the next most common anticonvulsant culprit agents, have more cases that triggered SJS/TEN after the 2000s. Antineoplastic-induced cases are skewed to the more recent half of the study period with the vast majority reported after the start of the 2000s. This upward publication trend in antineoplastics parallels the notable increase in the incidence of cancer internationally as well as the growing oncology literature during the study period (31). For all sub-categories, including systemic enzyme inhibitors and antimetabolites, nearly all cases were published after 2000. NSAIDs, specifically ibuprofen, similarly mirror the trend seen in antineoplastics; however, NSAIDs have triggered fewer reported cases of SJS/TEN in general. At large, these fluctuations in publishing trends may be indicative of changes in prescribing practices, incidences of various health conditions, and reporting biases.

### Drug culprits associated with Stevens-Johnson syndrome and toxic epidermal necrolysis

An overwhelming amount of research demonstrates that drugs are the primary causal agents, accounting for nearly 90% of SJS/TEN cases (7). This is consistent with the finding of the present study. We have compiled a comprehensive list of 379 drug culprit agents reported to be associated with SJS and TEN, which is more than most published SJS/TEN studies. Other studies, including Hsu et al. (1) did not study a similarly exhaustive list of medications despite their large sample sizes. The commonly reported medications (e.g., phenytoin, trimethoprim-sulfamethoxazole, carbamazepine) correspond to the list of highly suspected drugs associated with SJS/TEN in preexisting literature (6–8, 12, 32). Among all the drug classes, antibiotics, in particular sulfonamides, were reported to cause the highest number of TEN cases, and analgesics/anesthetics accounted for a higher proportion of SJS-TEN overlap cases. The remaining drug classes had no obvious differences in terms of the percentages among SJS, TEN, and SJS-TEN overlap cases.

Investigating immunosuppressive conditions and preconditions may reveal whether these conditions or related treatments amplify the risk of SJS/TEN. Risk factors, such as cancer, autoimmune disease, and infection, appear to be associated with SJS/TEN diagnosis in this study and prior research (1, 33, 34). Nearly a fifth of all cases reported having cancer prior to being diagnosed with SJS/TEN. Additionally, antineoplastics are one of the most frequently prescribed medications stated to cause SJS/TEN. Imatinib, methotrexate, lenalidomide, and nivolumab were among the most common antineoplastic agents listed as a causative drug. The increasing SJS/TEN cases among cancer patients suggests that the diseased cancer state and anticancer medication regimens may cause patients to be susceptible to severe cutaneous adverse reactions.

To a lesser extent, epilepsy and seizure disorders are a notable comorbidity, affecting nearly one in every ten cases. The high prevalence of epileptic disorders partially explains the significant number of anticonvulsants induced cases. At the same time, patients diagnosed with cancer who are treated with radiotherapy are often also treated with multiple medications, including anticonvulsants to preemptively abate seizures. While the occurrence of SJS/TEN in patients undergoing radiotherapy is rare, this condition has been frequently recognized in patients who are taking anticonvulsant drugs [i.e., phenytoin (35–46), carbamazepine (47), or antineoplastics (43, 48, 49)] while receiving cranial radiation.

In addition to cancer and epileptic disorders, approximately 5% of cases had a preexisting HIV infection, an established risk factor for SJS/TEN (1, 3, 11). Other studies noted a similar rate of 5−7% HIV cases among SJS/TEN cases, which is often higher than the controls for studies with a control group (7, 8). Data from the Nationwide Inpatient Sample from 2009 to 2012 also confirms that HIV/AIDs is one of the most common primary diagnoses for patients diagnosed with SJS/TEN (1). As Mockenhaupt et al. (8) suggest, HIV-associated cases have not significantly fluctuated over time as HIV incidence has stabilized. Still, as standard treatment has evolved, the causative agents associated with SJS/TEN have also changed. Of note, there is a preponderance of nevirapine-associated SJS/TEN cases in patients with HIV, accounting for 39% of all cases with HIV/AIDs. Additionally, nearly 12% of patients with HIV received trimethoprim-sulfamethoxazole, a common cause of adverse reactions in HIV patients (50) and one of the leading causes of SJS/TEN alone.

Beyond studying SJS/TEN through preconditions and risk factors, the compilation of case reports facilitated the identification of unique medication categories that are not often studied in relation to SJS/TEN including herbal medications and vaccines. Herbal medications/vitamins and vaccines are implicated as culprit agents in nearly 2% of cases. These drug categories are not frequently cited to cause SJS/TEN; however, in the case of herbal medications, the lack of cases may be due to underreporting in populations that are more likely to use herbal medications and not as likely to interface with allopathic medicine regularly. One study noted that 34% of people diagnosed with TEN in a burn center in Bangladesh took herbal medications and did not recall the medication name or its ingredients (51). Further analysis revealed that illiteracy and lack of financial resources influenced their use of herbal medications. The vague understanding of which herbal medications triggers SJS/TEN indicates that there are many unknowns associated with these medications and their true risk of causing SJS/TEN.

Unlike herbal medications, significant research has been performed to guarantee the overall safety of vaccines (52), yet common vaccines have also been linked to SJS/TEN, including the vaccines for influenza (53), smallpox, anthrax and tetanus (54), measles (55), the varicella-zoster virus (56), morbilli-parotitis-rubella (57), yellow fever (58), and rabies (59). Recently, COVID-19 vaccines were also reported to cause SJS/TEN (60, 61). Despite this potential risk, standard vaccines are not highly suspected to cause these reactions considering they account for 0.9% of cases. Moreover, relative to the sheer number of vaccines distributed annually, patients with vaccine-induced SJS/TEN represent a very small percentage of all vaccine recipients (62). However, these cases are difficult to validate as some probable cases were ill prior to receiving the vaccine or concomitantly taking other medications. All the same, it cannot be ruled out that SJS/TEN is a rare but possible adverse reaction for a small percentage of vaccine recipients.

Because many medical treatments involve multiple medications, it is difficult to determine whether a specific medication alone caused SJS/TEN without controlling for concomitant therapies (63). Approximately 16.6% of all cases were exposed to more than one medication at the time of the diagnosis and in those cases, it may be difficult to understand the influence of drug interactions. A medication that demonstrates the confounding effect of multiple drug therapies is the anticonvulsant valproate. Valproate was identified to cause 1.5% of SJS/TEN cases, suggesting it is a probable culprit agent that may trigger SJS/TEN. Yet 75% of patients receiving valproate were receiving other medications, particularly other anticonvulsants. Prior research reveals that valproate extends the half-life of lamotrigine such that lamotrigine persists in the body longer (64). Thus, while valproate alone has little to no significant risk of SJS/TEN, it increases the likelihood of an adverse reaction like SJS/TEN when interacting with specific medications (8, 64). With respect to cases involving antineoplastics, it is common to prescribe other medications in addition to antineoplastics, including anticonvulsants or antibiotics, which are also strongly associated with an SJS/TEN diagnosis. Several of the cases reporting more than one causative agent are patients with cancer, suggesting that patients with cancer may be at greater risk due to receiving a combination of highly suspected culprit agents that may interact and heighten the risk of SJS/TEN (39, 42, 43, 65).

### Non-medication culprits associated with Stevens-Johnson syndrome and toxic epidermal necrolysis

Non-drug allergens are reportedly associated with SJS/TEN in 12% of cases in the present study, among which, more than half implicated infections. Three-quarters of the cases with infections as the culprit agent triggered an SJS diagnosis, indicating a strong association between SJS and infections. This link has also been confirmed in other studies (1, 66). Dissimilarly, a vast majority of TEN cases are associated with a medication culprit agent (67). Within our data, 19% of all cases had an infection as a preexisting condition, and at least 2% were confirmed mycoplasma pneumoniae infections. Likewise, infections, specifically mycoplasma pneumoniae infections, were also classified as a non-drug allergen for about 6.4% and 4.2% of all cases, respectively. This discrepancy between how many reports identified the infection as a precondition or as a causative agent indicates that the exact causal mechanism of infections remains unknown (1). It is possible that the antibiotics or other medication used to treat the infection were the true causative agents. However, there are several case reports that did not identify any potential medication that could serve as a causative agent (68–70). Additionally, some research groups have suggested that mycoplasma pneumoniae is more likely to trigger erythema multiforme (EM) and not SJS/TEN. While EM was previously regarded to fall along the same spectrum of severe cutaneous reactions, EM and severe cutaneous reactions such as SJS/TEN have separate diagnostic criteria at present (71). Ultimately, the relationship between infections and SJS/TEN requires further exploration, and understanding the shared characteristics of cases with non-drug allergens will be invaluable in identifying potential risk factors for SJS/TEN or similar severe cutaneous reactions beyond common causative medications.

### Limitations of using case reports from literature to study Stevens-Johnson syndrome and toxic epidermal necrolysis

In general, algorithms that assessed drug causality were rarely reported in the case reports. Therefore, in many cases, the actual causative agent may be a probable but not definite cause for SJS/TEN. Similarly, there is uncertainty surrounding the true causative agents in several studies using EHR or registry database data (4, 9, 72). In addition to multiple drug interactions (7, 73, 74), increased dosage of a medication may also trigger SJS/TEN (8, 75). It is also unclear how many SJS/TEN cases are truly caused by non-drug allergens, considering 7.6% of cases solely implicate non-drug allergens. Furthermore, validating diagnoses of SJS/TEN can be challenging (39), and the definition of SJS/TEN has changed over time. This might result in the inclusion of some EM cases in our analysis inadvertently due to the author’s assessment, particularly with cases reported prior to 2000 when the diagnostic criteria were less defined (2, 76). Due to the retrospective nature of our study, we could not re-evaluate the case diagnosis, and only about half of the SJS/TEN case reports indicated confirmation from pathology results.

By design, data collected from case reports are not generalizable nor can we make causal inferences from case reports, unlike other evidence-based study designs. Although our study reviewed a large number of SJS/TEN cases, there was no way to form a control group for comparison to identify differences that result from an SJS/TEN diagnosis. Also, while our data captures comprehensive patient information, our compiled data cannot be used to infer the epidemiology of SJS/TEN. With publication biases, some cases are more likely to be reported than others, impacting the generalizability of our findings.

Studies that rely on database or registry data may be more capable of overcoming certain reporting biases. For instance, Fukasawa et al. (16) used a large-scale employee claims database that includes longitudinal inpatient, outpatient, and pharmacy information for all employees receiving national coverage to approximate the true and relative risk of SJS/TEN in the Japanese population (77). However, selection bias may still be involved from excluding cases that arise from uncommon medications or causes that are not recorded in the database (9, 62). Also, not all studies take precautions to validate cases or define a control population (9). Despite lacking a control group and being subject to publication biases, significant results from our data remain consistent with data extracted from large-scale databases and registries.

Additionally, missing data due to a lack of standardized criteria that promoted complete, detailed reports made it difficult to detect associations between patient characteristics and SJS/TEN. Such variable level of detail in each case report complicated the annotation and analysis process. For instance, a majority of cases did not report race or ethnicity, inhibiting us from uncovering associations between race and incidence of SJS/TEN diagnosis for certain medications (6, 34, 78). Also, case reports used different terms to refer to the same medication. Because case reports are published according to differing journal-specific standards (79), a broader quality metric does not exist to ensure high quality data reporting. Still, nearly half of all included cases contained pathology results, and approximately 66% contained photographs as reference, indicating that reports have the potential to be very comprehensive and provide invaluable clinical insight. Establishing a quality measure can help ensure the clinical utility of case reports and may minimize publication bias.

Despite these shortcomings, case reports are a rich source of detail regarding the etiology, clinical courses, and potential treatments of SJS/TEN. The information extracted from case reports can shape clinical guidelines for providing care for prospective SJS/TEN patients and, ultimately, enhance our medical understanding these reactions.

## Conclusion

Our study assembled a large, unique set of SJS/TEN cases from the literature and provided an extensive list of potential causative agents associated with SJS/TEN. By identifying differences across the disease spectrum and trends across individual case reports, this research builds a more holistic understanding of SJS/TEN, extracting information from seminal research in the field and validating trends observed in prior studies. For future research, it is necessary to understand the distinct impact of individual medications on SJS/TEN progression and how culprit agents differ in various populations. The sheer abundance of case reports and the level of detail therein will likely support efforts to address these next steps in SJS/TEN research.

## Data availability statement

The raw data supporting the conclusions of this article will be made available by the authors, without undue reservation.

## Author contributions

LW had full access to all the data in this study, took responsibility for the integrity of the data and the accuracy of the data analysis, and did the concept and design. LW, SV, FB, Y-CL, CO, and SS did the acquisition, analysis, or interpretation of data. LW and SV drafted the manuscript and performed the statistical analysis. KB, EP, and LZ obtained the funding. LW and LZ contributed to the administrative, technical, or material support. LW, KB, EP, and LZ supervised the data. All authors critically revised the manuscript for important intellectual content.

## Abbreviations

SJS: Stevens-Johnson syndrome
TEN: Toxic epidermal necrolysis
HIV: Human immunodeficiency virus
EHR: Electronic health records
EM: Erythema multiforme
NSAIDs: Non-steroidal anti-inflammatory drugs.
